# Deformation of Copper Nanowire under Coupled Tension–Torsion Loading

**DOI:** 10.3390/nano12132203

**Published:** 2022-06-27

**Authors:** Hongquan Lu, Bin Dong, Junqian Zhang, Chaofeng Lü, Haifei Zhan

**Affiliations:** 1 College of Civil Engineering and Architecture, Quzhou University, Quzhou 324000, China; 2 College of Civil Engineering and Architecture, Zhejiang University, Hangzhou 310058, China; 22012088@zju.edu.cn (B.D.); lucf@zju.edu.cn (C.L.); 3 Shanghai Institute of Applied Mathematics and Mechanics, School of Mechanics and Engineering Science, Shanghai University, Shanghai 200444, China; jqzhang2@shu.edu.cn; 4 Shanghai Key Laboratory of Mechanics in Energy Engineering, Shanghai 200444, China; 5 Faculty of Mechanical Engineering & Mechanics, Ningbo University, Ningbo 315211, China; 6 School of Mechanical, Medical and Process Engineering, Queensland University of Technology (QUT), Brisbane, QLD 4001, Australia

**Keywords:** nanowires, yielding, torsion, tension, dislocation, molecular dynamics simulation

## Abstract

Metallic nanowires (NWs) are essential building blocks for flexible electronics, and experience different deformation modes due to external mechanical loading. Using atomistic simulations, this work investigated the deformation behavior of copper nanowire under coupled tension–torsion loading. A transition in both yielding pattern and dislocation pattern were observed with varying torsion/tension strain ratios. Specifically, increasing the torsion/tension strain ratio (with larger torsional strain) triggered the nucleation of different partial dislocations in the slip system. At low torsion/tension strain ratios, plastic deformation of the nanowire was dominated by stacking faults with trailing partial dislocations pinned at the surface, shifting to two partial dislocations with stacking faults as the strain ratio increases. More interestingly, the NW under tension-dominated loading exhibited a stacking fault structure after yielding, whereas torsion-dominated loading resulted in a three-dimensional dislocation network within the structure. This work thus suggests that the deformation behavior of the NW varies depending on the coupled mechanical loading, which could be beneficial for various engineering applications.

## 1. Introduction

Metallic nanowires (NWs) are essential building blocks for various high-end applications, such as nanoelectromechanical systems (NEMS), flexible electronics [1], sensors [2], and solar cells [3,4]. Especially for flexible electronics [5,6], NWs experience different mechanical deformation modes, such as bending, torsion, and tension [7]. Therefore, understanding the mechanical behavior of metallic NWs is of great importance. There are extensive works investigating the mechanical behaviors of different types of metallic NWs through experiments and atomistic simulations [8,9,10,11,12]. For instance, face-centered-cubic (FCC) NW is reported to exhibit phase transformation phenomena [13] or structural transformation [14], the shape memory effect [15,16,17], and rubber-like behavior [18], and the failure feature of NW changes from ductile to brittle as its length increases [19]. Various factors have been found to impact the mechanical behavior of metal nanowires [20,21], such as size [9], grain size [11,22], strain hardening [23], strain rate [24], twinning [25], and surface stress [26,27,28]. In addition to perfect single crystalline NW, many researchers have investigated the deformation process of twinned NWs [22,23,29,30] and reported on NWs with different microstructural features that exhibit excellent mechanical properties [11,20,31].

The literature shows that the yielding and subsequent plastic deformation of FCC NWs under tensile loading are attributed to slip via partial dislocations, full dislocations, and twinning [8,29,32,33,34,35]. In addition to tensile deformation, a substantial number of works have exploited the deformation behavior of FCC NWs under compression [36,37,38], torsion [30,39,40,41,42,43], bending [44,45,46], and vibration [47,48]. The torsional detwinning domino has been reported to result in giant rotational deformation of Cu nanorods without localized failure [49]. Specifically, <110> orientated NWs have been found to exhibit homogeneous deformation under torsion through dislocations nucleated along their axis, while <111> and <100> oriented NWs have been found to deform through the formation of twist boundaries [41,50]. Despite extensive works on the mechanical properties of NWs, their deformation behavior under coupled mechanical loading have been less frequently investigated. Considering their broad applications in electronics, it is of great interest to assess how NWs deform under coupled mechanical stimuli.

To that end, this work systematically investigated the deformation of <100> orientated monocrystalline Cu NW under coupled tension–torsion loading via molecular dynamics (MD) simulations. It was found that NW failure is triggered by partial dislocation nucleated at its surface, while the partial dislocation type changes as the torsion/tension strain ratio is varied.

## 2. Methods

This paper considered cylindrical Cu NW with an orientation of <100> and a diameter of about 20*a*, where *a* is the lattice constant. The sample had a length of 40*a*, with a total of 55,491 atoms. The commonly used embedded atom method (EAM) potential was used to describe Cu–Cu atomic interactions [51]. The conjugate gradient minimization method was first applied to optimize the NW, and the specimen was equilibrated under the canonical ensemble using a Nosé–Hoover thermostat under 300 K [52,53] for 50 ps. During the structural relaxation, no periodic boundary conditions were applied. All simulations were performed using the open-source package LAMMPS [54], and a time step of 1 fs was used for the all simulations.

The effective coupled tension–torsion strain is represented by the loading parameter ξ with $\dot{\xi }=\sqrt{{\dot{\varepsilon }}^{2}+{\dot{\gamma }}^{2}}$, where ε and γ are the axial normal strain and the shear strain at the surface, respectively. The normal strain is engineering tensile strain calculated as $\varepsilon =\Delta L/L_{0}$, with ΔL and $L_{0}$ representing the length change and the initial length of the NW, respectively. The shear strain is calculated from $\gamma =\phi R/L_{0}$, where *R* is the radius and ϕ is the twist angle. The torsion/tension strain ratio is defined as $k_{\varepsilon }=\gamma /\varepsilon$, and $k_{\varepsilon }=0$ and $k_{\varepsilon }=\infty$ correspond to the uniaxial tension and the pure torsion scenario, respectively. The twist angle and the axial displacement are related to the loading parameter and the proportional loading angle, with $\Delta L=L_{0}\xi /\sqrt{1+k_{\varepsilon }^{2}}$ and $\Delta \phi =\left(L_{0}\xi k_{\varepsilon }\right)/\left(R\sqrt{1+k_{\varepsilon }^{2}}\right)$, respectively. Thus, for any $k_{\varepsilon }$, the axial deformation and twist angle can be calculated accordingly. Two unit cells at each end were treated as a rigid body to apply the coupled load, and the coupled load was imposed on the NW in a quasi-static manner; that is, the coupled load was applied to the sample step by step and the sample was relaxed under the canonical ensemble using the Nosé–Hoover thermostat under 300 K [52,53] for 50 ps between each loading step. Here, we adopted a constant strain increment of Δξ = 0.0005 for all tests, resulting in a length change less than 0.25% per step. A summary of the coupled tension–torsion loading parameters is provided in Table 1. The overall tensile stress (σ) of the NW was evaluated by the virial stress averaged over the whole NW, while the overall torque ($T_{z}$) was calculated by the virial torque [50], i.e., $T_{z}=\frac{1}{L}\sum_{i}\omega_{i}\left(\sigma_{yz}^{i}x^{i}-\sigma_{xz}^{i}y^{i}\right)$. Here, z is the axial direction of the NW. L is the sample length, $\omega_{i}$ is the atom volume, $x^{i}$ and $y^{i}$ are the lateral coordinates; and $\sigma_{yz}^{i}$ and $\sigma_{xz}^{i}$ represent the two shear stress components for the ith atom. The nominal shear stress (τ) at the surface is computed by $\tau =2T_{z}/\left(\pi R^{3}\right)$.

## 3. Results and Discussion

**Figure 1:**
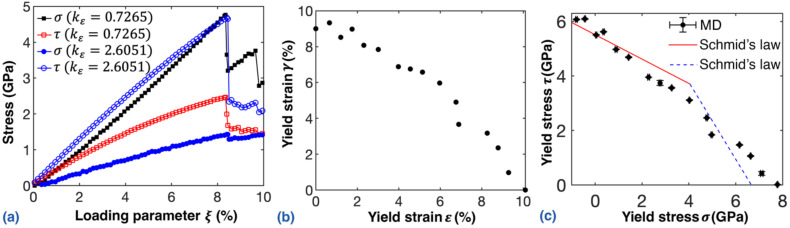
The mechanical properties under different torsion/tension strain ratios: (**a**) stress-loading parameter curves of the Cu NW with two torsion/tension strain ratios $k_{\varepsilon }$; (**b**) yielding strain pattern; and (**c**) yield stress pattern. The solid and dashed lines are fitted following Schmid’s law using the nonlinear least square method.

First, we examined the deformation of the NW under different torsion/tension ratios by varying the arctan $\left(k_{\varepsilon }\right)$ from 0 to 90°. Figure 1a compares the normal stress and the nominal shear stress at the surface for two torsion/tension strain ratios, i.e., $k_{\varepsilon }=0.7265$ (Test 6) and $k_{\varepsilon }=2.6051$ (Test 11), corresponding to a tension-dominated and a torsion-dominated scenario, respectively. In general, the Cu NW exhibits a similar stress–strain profile as that under pure tension or torsion. The normal stress or nominal shear stress increases continuously until reaching a critical magnitude, and afterwards the stress experiences a sudden drop. Such a stress profile is observed in all examined torsion/tension ratios. Compared with the yield strain for either tension [32] or torsion in the literature, the NW experiences earlier failure due to the coupled mechanical loading. For the tensile-dominated scenario ($k_{\varepsilon }=0.7265$), the Cu NW shows a maximum normal stress and nominal shear stress of about 4.76 GPa and 2.47 GPa, respectively. The corresponding critical loading parameter is about 8%, which is analogous to the yield strain. In comparison, NW under the torsion-dominated scenario ($k_{\varepsilon }=2.6051$) exhibits a maximum normal stress and nominal shear stress of about 1.43 GPa and 4.69 GPa, respectively. A slightly larger critical loading parameter of 8.2% is observed. These results suggest that the Cu NW exhibits different mechanical properties when the torsion/tension strain ratio varies.

Figure 1b illustrates the yield pattern of the NW at different torsion/tension strain ratios. According to the simulation results, the critical loading parameter ξ varies from 7.5% to 9.1%. For pure torsion, the yielding point is about 9.0%, which is about 10.1% for pure tension scenario. As expected, the yielding point for shear or tensile strain varies when the loading strain ratio changes, and a higher shear yield strain is accompanied by a lower tensile yield strain. Similarly, the shear stress and the tensile stress can be extracted at the yield point. As plotted in Figure 1c, the shear stress and tensile stress at the yield point share a similar patten to that of the yield strain. It is notable that slight compressive stress is observed for the pure torsion test and the case close to pure torsion (Tests 15 and 16 in Table 1). This observation can be attributed to the Poisson’s effect. Strong radial compression appears during pure torsion [55], which should lead to the slight stretching of the NW in the axial direction with a positive Poisson’s effect. However, the sample length was kept constant during our simulation; thus, the NW exhibits compressive stress.

**Figure 2:**
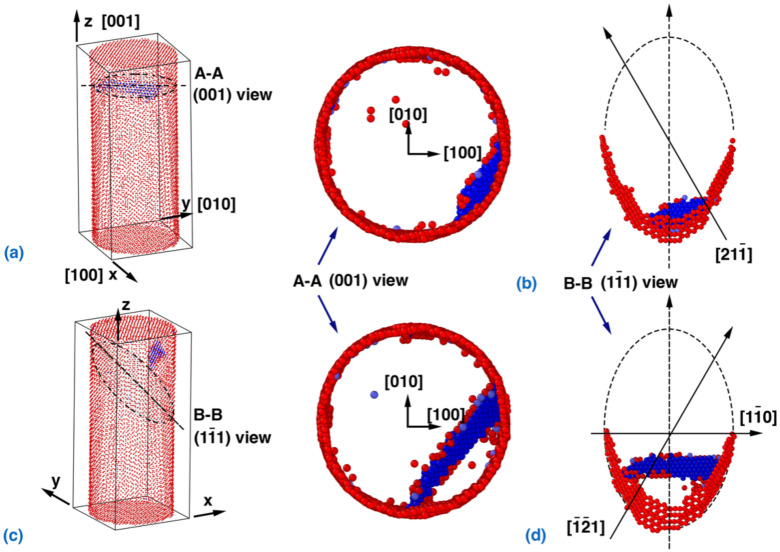
Dislocation nucleation and slip in the NW with a torsion/tension strain ratio of 2.6051. Development of the dislocation: (**a**) cross-sectional view of the nucleation and (**b**) the (1$\bar{1}$1) plane of the nucleation. Below, a view of the dislocation formed by the trailing partial dislocations: (**c**) cross-sectional view and (**d**) the (1$\bar{1}$1) plane. Atoms with the Ackland–Jones parameter (AJP) [56] equal to 0 and 3 are visualized in order to distinguish the atoms in the surface and partial dislocation/stacking faults, respectively. Specifically, AJP=0 highlights the surface atoms (colored in red), and AJP=3 highlights the HCP atoms (colored in blue). The FCC atoms with AJP=2 are not shown in the figure for clarity.

In order to explore the deformation process, the atomic configurations of the NW under coupled loading in a torsion-dominated ($k_{\varepsilon }=2.6051$) and tension-dominated ($k_{\varepsilon }=0.7265$) scenario were examined. As shown in Figure 2a, a $\left(1\bar{1}1\right)/\left[21\bar{1}\right]$ single partial dislocation nucleates from the free-surface of the NW, leading to a sudden stress drop event. The location of the leading partial dislocation nucleation is near the vertex of $\left(1\bar{1}1\right)$ elliptical slip planes (Figure 2b). From Figure 2c,d, the $\left(1\bar{1}1\right)/\left[\bar{1}\bar{2}1\right]$ trailing partial dislocation nucleates near the vertex of $\left(1\bar{1}1\right)$ elliptical slip plane, leading to the formation of the $\left(1\bar{1}1\right)/\left[1\bar{1}0\right]$ perfect edge dislocation. The two partial dislocations and the associated stacking faults propagate into the interior of the NW.

The maximum resolved shear stress (MRSS) at or near the surface can be used to explain the yielding behavior of the NW. There are three potential slip systems on each of the four equivalent slip planes. The resolved shear stresses (RSS) in these three slip systems at the free surface vary along the circumference of the NW. For the torsion/tension ratio $k_{\varepsilon }=2.6051$, the MRSS on the $\left(1\bar{1}1\right)$ elliptical slip plane was found to be in the $\left[21\bar{1}\right]$ and $\left[\bar{1}\bar{2}1\right]$ slip systems, equal to $\tau_{\left(1\bar{1}1\right)/\left[\bar{1}\bar{2}1\right],\left[21\bar{1}\right]}=\sqrt{2}\left(\sigma +\sqrt{5}\tau \right)/6$ at sites rotating from the two vertex points of the ellipse by 30° (Figure 2b). This observation suggests that the RSS can correctly reveal both the orientation of the nucleated partial dislocation as well as its nucleation location. It is notable that the $\left(1\bar{1}1\right)/\left[21\bar{1}\right]$ nucleation represents a random choice among eight identical slip systems/sites (two for each of the four equivalent planes) when the RSS is the same.

**Figure 3:**
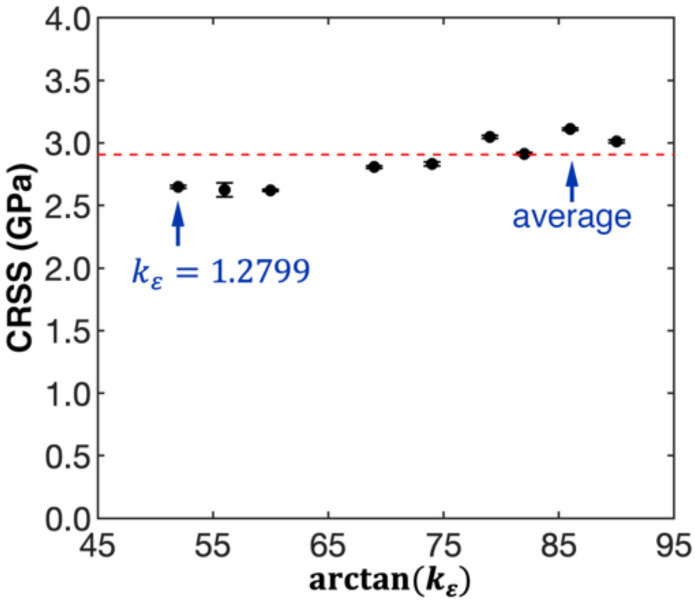
Variation of the critical resolved shear stress (CRSS) when the torsion/tension strain ratio changes ($k_{\varepsilon }\geq 1.2799$).

According to the atomic configurations, when $k_{\varepsilon }\geq 1.2799$ (Tests 8 to 16 in Table 1), the Cu NW exhibits a uniform failure mechanism that is triggered by the nucleation of the $\left(1\bar{1}1\right)/\left[21\bar{1}\right]]$ (or identical) single partial dislocation. Meanwhile, the slip system and the location of the leading partial dislocation is the same. This observation aligns well with the MRSS, which appears in the $\left(1\bar{1}1\right)/\left[\bar{1}\bar{2}1\right]$ direction. Based on the RSS definition, the critical resolved shear stress (CRSS) can be computed using the yield stress obtained from the coupled tension–torsion loading simulation (Figure 1c). As shown in Figure 3, the estimated CRSS fluctuates around 2.91 GPa for $k_{\varepsilon }\geq 1.2799$, which exhibits weak correlation with the torsion/tension strain ratio.

**Figure 4:**
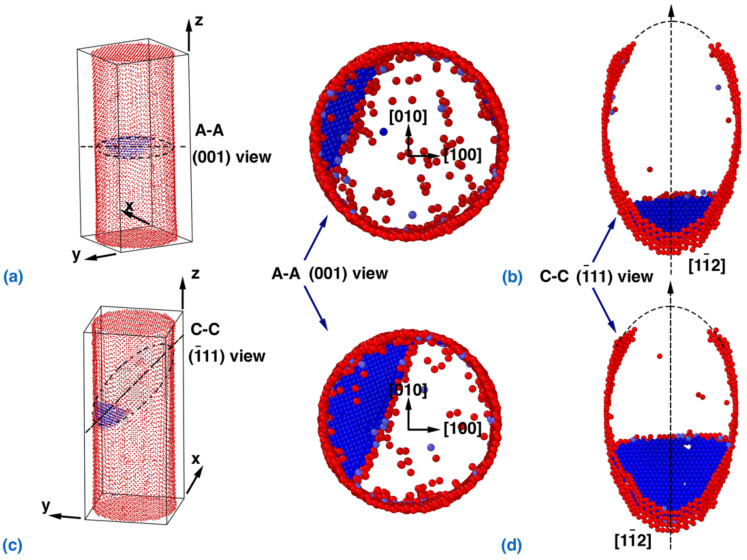
Dislocation nucleation and slip in the NW with a torsion/tension strain ratio of 0.7265. View of the dislocation nucleation from: (**a**) cross-sectional view, i.e., the (001) plane, and (**b**) the ($\bar{1}$11) plane. Below, a view of the propagation of the partial dislocation that forms stacking faults with trailing partial dislocations pinned at the surface; (**c**) cross-sectional view and (**d**) the (111) plane. Atoms with the Ackland–Jones parameter (AJP) [56] equal to 0 and 3 are visualized in order to distinguish the atoms in the surface and partial dislocation/stacking faults, respectively.

Compared with the torsion-dominated loading scenario in Figure 2, the Cu NW exhibits different failure mechanisms under the tension-dominated scenario ($k_{\varepsilon }=0.7265$). According to Figure 4a, the $\left(\bar{1}11\right)/\left[1\bar{1}2\right]$ partial dislocation nucleates at the surface of the NW, which triggers its failure. With increasing strain, the partial dislocation propagates into the interior of the NW (Figure 4b), and no trailing partial dislocation is observed. Theoretically, the RSS in the $\left(\bar{1}11\right)/\left[1\bar{1}2\right]$ slip system can be calculated from $\tau_{\left(\bar{1}11\right)/\left[1\bar{1}2\right]}=\sqrt{2}\left(2\sigma +\sqrt{2}\tau \right)/6$, and the dislocation nucleation occurs randomly in the four identical slip systems. It is found that the Cu NW exhibits a same failure mechanism for the torsion/tension strain ratio $k_{\varepsilon }$ between 0 and 1.0 (Tests 1 to 7 in Table 1).

Specifically, the nucleation site of the $\left(\bar{1}11\right)/\left[1\bar{1}2\right]$ partial dislocation cannot be explained by the MRSS alone, as the RSS in the $\left(\bar{1}11\right)/\left[1\bar{1}2\right]$ slip system reaches the maximum at the end points of the minor axis of the elliptical slip plane (for $k_{\varepsilon }$ between 0 and 1.0). According to the literature, this phenomenon can be explained from the perspective of the energy barrier [57]. In fact, the energy barrier is lower at the vertex than that at end points of the minor axis, as the dislocation loop around the vertex of the elliptical slip plane is shorter than that around the end points of the minor axis. Thus, the CRSS is regarded as the upper bound for the yielding of the Cu NW under loading with $k_{\varepsilon }$ between 0 and 1.0. Recall that in Figure 1c, the yield stresses obtained from MD simulations fit well with the CRSS predicted by Schmid’s law, suggesting that Schmid’s law can be used to predict the yielding strength of the NW under coupled tension–torsion loading.

**Figure 5:**
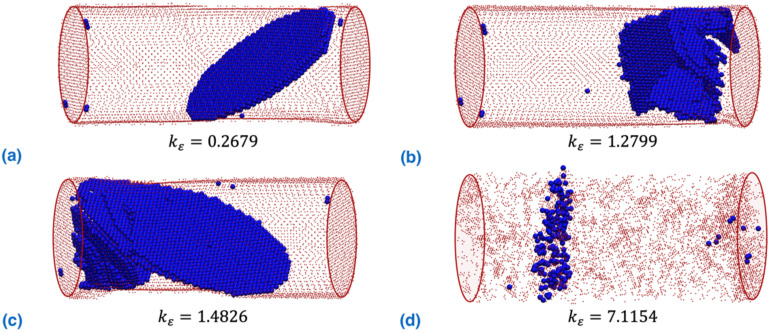
Dislocation patterns of NW after yielding before resumption of stress increase for different torsion/tension strain ratios: (**a**) $k_{\varepsilon }=0.2679$; (**b**) $k_{\varepsilon }=1.2799$; (**c**) $k_{\varepsilon }=1.4826$; (**d**) $k_{\varepsilon }=7.1154$. Atoms with the Ackland–Jones parameter (AJP) [56] equal to 0 and 3 are visualized in order to identify the atoms in the surface and partial dislocation/stacking faults, respectively.

Interestingly, Figure 5 compares the dislocation patterns of the NW after the first sudden stress drop event (before the stress resumes the increasing trend) for four different torsion/tension strain ratios. As can be seen, the NW exhibits a completely different dislocation pattern. Under the tension-dominated scenario ($k_{\varepsilon }=0.2679$, Figure 5a), well-aligned stacking faults with trailing partial dislocations pinned at the surface are generated. With the increasing torsion component, more partial dislocations are nucleated (Figure 5b,c), which form a three-dimensional dislocation network (referred to in the literature as a twist boundary [41,50]). For instance, the NW under $k_{\varepsilon }=7.1154$ shows a three-dimensional dislocation network without a clear formation of stacking faults, with trailing partial dislocations pinned at the surface. Overall, the plastic deformation of the NW experiences a transition from a stacking fault-dominated scheme to a dislocation network-dominated scheme when the torsion/tension strain ratio increases.

## 4. Conclusions

In summary, this work systematically investigated the yielding pattern of <100> oriented Cu NW under coupled torsion–tension loading. Our simulations show that a transition of the slip system for dislocation nucleation occurs when the torsion/tension ratio increases, and more partial dislocations are active when the torsion component increases. Yielding of the NW is provoked by the nucleation of partial dislocation of the $\left(\bar{1}11\right)/\left[1\bar{1}2\right]$ type with lower torsion/tension strain ratios (tension-dominated loading), changing to partial dislocation of the $\left(1\bar{1}1\right)/\left[21\bar{1}\right]$ type with higher torsion/tension ratios (torsion-dominated loading). After yielding, the NW under tension-dominated loading exhibits stacking fault plastic deformation, whereas torsion-dominated loading results in a three-dimensional dislocation network within the structure. This work provides a comprehensive understanding of the deformation behavior of Cu NW under coupled mechanical loading, which could be beneficial for the failure prediction of NWs in different engineering application scenarios.

## Figures and Tables

**Table 1:** A summary of the coupled tension–torsion loading settings for different tests.

Test	$k_{\varepsilon }$	$arctan\;\left(k_{\varepsilon }\right)$	ΔL/2 (a)	Δϕ/2 (°)
1	0	0	0.01	0
2	0.1051	6	0.0099	0.0060
3	0.2679	15	0.0097	0.0148
4	0.3839	21	0.0094	0.0205
5	0.5317	28	0.0088	0.0269
6	0.7265	36	0.0081	0.0337
7	1.0000	45	0.0071	0.0405
8	1.2799	52	0.0062	0.0451
9	1.4826	56	0.0056	0.0475
10	1.7321	60	0.0050	0.0496
11	2.6051	69	0.0036	0.0535
12	3.4874	74	0.0028	0.0551
13	5.1446	79	0.0019	0.0562
14	7.1154	82	0.0014	0.0567
15	14.3007	86	0.0007	0.0572
16	∞	90	0	0.0573

Note: $k_{\varepsilon }=0$ and $k_{\varepsilon }=\infty$ correspond to the pure tension and pure torsion, respectively.

## Data Availability

The data that support the findings of this study are available from the corresponding authors on reasonable request.
